# Ondansetron HCl Microemulsions for Transdermal Delivery: Formulation and *In Vitro* Skin Permeation

**DOI:** 10.5402/2012/428396

**Published:** 2012-06-19

**Authors:** Jadupati Malakar, Amit Kumar Nayak, Aalok Basu

**Affiliations:** ^1^Department of Pharmaceutics, Bengal College of Pharmaceutical Science and Research, Durgapur 713212, India; ^2^Department of Pharmaceutics, Seemanta Institute of Pharmaceutical Sciences, Mayurbhanj 757086, India

## Abstract

Ondansetron HCl delivery through oral route suffers due to its low bioavailability due to first-pass metabolism. Therefore, the microemulsion-based transdermal delivery may be a better substitute for it. The pseudoternary phase diagrams were constructed to determine compositions of microemulsions, and ondansetron HCl microemulsions for transdermal delivery were developed using isopropyl myristate or oleic acid as the oil phase, Tween 80 as the surfactant, and isopropyl alcohol as the cosurfactant evaluated for *in vitro* skin permeation through excised porcine skin. The *in vitro* skin permeation from these formulated microemulsions was sustained over 24 hours. The microemulsion F-8 (contained 10% of isopropyl myristate as oil phase, 8% of aqueous phase, and 82% of surfactant phase containing Tween 80 and isopropyl alcohol, 3 : 1) showed the highest permeation flux of 0.284 ± 0.003 *μ*g/cm^2^/hour. All these microemulsions followed the Korsmeyer-Peppas model (*R* ^2^ = 0.971  to  0.998) with non-Fickian, “anomalous” mechanism over a period of 24 hours.

## 1. Introduction 

Ondansetron HCl is a 5-HT_3_ receptor antagonist indicated for the treatment and/or prophylaxis of postoperative or chemotherapy- or radiotherapy-induced emesis [1]. It is well absorbed and undergoes first-pass metabolism [2]. The oral bioavailability of ondansetron HCl is almost 59%, and peak plasma about 0.03–0.04 *μ*g/mL is obtained after 1.5 to 2 h of administration [3]. As administering drug through the transdermal route avoids hepatic first-pass metabolism, thus, the delivery of ondansetron HCl to the systemic circulation via the transdermal route would improve its bioavailability. 

In the previous literature, various attempts of transdermal delivery of ondansetron HCl were also reported [4–7]. Transdermal delivery has many advantages over conventional modes of drug administration as it avoids hepatic first-pass metabolism and improves patient compliance [8]. In addition, it is simple to terminate the therapy, if any adverse or undesired effect occurs. However, skin is a natural barrier, and only a few drugs can penetrate the skin easily and in sufficient quantities to be effective. In recent years, numerous drug penetration enhancement techniques were studied through the transdermal route [9, 10]. Among them, one of the most promising techniques for enhancement of transdermal drug permeation is the microemulsion technique [11–16]. Microemulsions as colloidal carriers are one of the promising systems that have nowadays attracted the main interest in penetration enhancement. They are optically isotropic, transparent, and thermodynamically stable homogeneous solutions of oil and water, stabilized by addition of a surfactant and usually a cosurfactant [13, 14]. Due to their special features, microemulsions offer several advantages for the pharmaceutical use, such as enhanced drug solubilisation, good thermodynamic stability, ease of preparation, low viscosity, high drug loading capacity, and small droplet size, which make them promising as a drug delivery tool [17, 18]. As a vehicle for transdermal systems, microemulsions can increase the local or systemic delivery of drugs by different mechanisms [15, 18, 19]. First, their composition and structure enable them to incorporate a greater amount of drug due to the high solubilizing capacity with increased thermodynamic property toward the skin than various conventional topical formulations. Second, the diffusional barrier of the skin may be modified depending on the composition of microemulsion system. Third, the surfactants and cosurfactants used in microemulsions may reduce diffusional barriers by acting as penetration enhancers. In the current investigation, the transdermal delivery system of ondansetron HCl through microemulsions was investigated. Ondansetron HCl microemulsions for transdermal delivery, containing isopropyl myristate or oleic acid as the oil phase, Tween 80 as the surfactant, and isopropyl alcohol as the cosurfactant, were formulated and evaluated. 

## 2. Materials and Methods

### 2.1. Materials

Ondansetron HCl (B.S Traders Pvt. Ltd., India), isopropyl myristate (Merck Specialties Pvt. Ltd. India), oleic acid (Qualigens Fine Chemicals, India), isopropyl alcohol (Qualigens Fine Chemicals, India), and Tween 80 (Merck Specialties Pvt. Ltd. India) were used. All chemicals and reagents used were of analytical grade. 

### 2.2. Construction of Pseudoternary Phase Diagram

**Figure 1:**
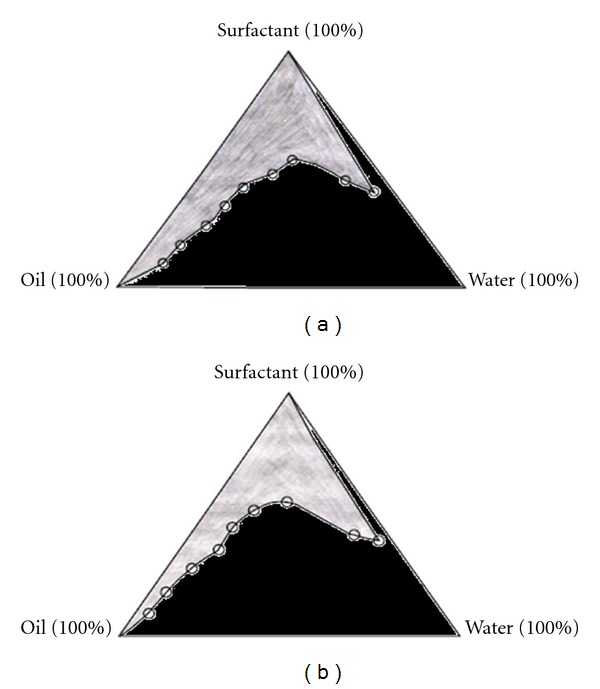
Pseudoternary phase diagrams of (a) oil (isopropyl myristate), surfactant (Tween 80 and isopropyl alcohol, in ratio 3 : 1), and aqueous phases (b), (a) oil (oleic acid), surfactant (Tween 80 and isopropyl alcohol, in ratio 3 : 1) and aqueous phases. The nondarken regions represent microemulsion zones.

Microemulsions were prepared by using conventional titration method. The oil (oleic acid or isopropyl myristate) and aqueous phases were first combined with the surfactant (Tween 80). Cosurfactant (isopropyl alcohol) was added gradually with magnetic stirring at room temperature until the system was transparent. Transparent, single-phase formulations were indicative of stable microemulsions. Microemulsions were allowed to equilibrate with gentle magnetic stirring for 15 minutes. These microemulsions were then titrated with water using a micropipette at room temperature. Then, these were stirred vigorously for a sufficient length of time and end point (onset of turbidity or phase separation) was visually monitored against a dark background by illuminating the samples with a white light. The experiments were performed in triplicate to check reproducibility. From the end point composition of titrated samples, the mass percent composition of the components like oil, surfactant, and water was calculated and plotted on triangular coordinates to construct a pseudoternary phase diagram [16]. The pseudoternary phase diagrams were constructed to determine the composition of an aqueous phase, an oil phase, and a surfactant: cosurfactant (3 : 1) phase that will yield microemulsions (transparent solutions) at room temperature which were represented in the nondarkened area of the following diagrams (Figure 1). 

### 2.3. Preparation of Ondansetron HCl Microemulsions

From the microemulsion regions in the pseudoternary phase diagram, the eight different formulas for the development ondansetron HCl W/O microemulsions were selected as shown in Table 1. Ondansetron HCl W/O microemulsions were prepared by mixing candesartan cilexetil to the mixture of oleic acid, Tween 80, and isopropyl alcohol. An appropriate amount of distilled water was added to the mixture drop by drop while with vigorous stirring using a magnetic stirrer (Remi Motors, India) until the transparent microemulsions were produced. These microemulsions were allowed to equilibrate with gentle magnetic stirring for 15 minutes, and then these were passed through Whatman filter paper (no. 40). All formulations contained 20 mg of ondansetron HCl.

### 2.4. Preparation of Skin for * In Vitro * Permeation Studies. 

Porcine skin was used for the permeation studies of the prepared formulations. The skin was obtained from the slaughterhouse after sacrificing the animal within 1 hour. Then the hair was removed from the upper portion of the ear skin using an animal hair clipper, and subsequently full thickness of the skin was harvested. The fatty layer, adhering to the dermis side, was removed by surgical scalpel. Finally, these excised skins were thoroughly rinsed with distilled water and packed in aluminum foils. The skin samples were stored at −20°C and used within a week. 

### 2.5. * In Vitro * Skin Permeation Study by Franz Diffusion Cell. 

The permeation of formulated all ondansetron HCl microemulsions was carried out using Franz diffusion cell. The cell consists of two chambers, the donor and the receptor compartment with a diffusion area of 0.785 cm^2^. The donor compartment was open at the top and was exposed to atmosphere. The excised porcine skin was mounted between the compartments of the diffusion cell with stratum corneum facing the donor compartment and clamped into position. Magnetic stirrer bars were added to the receptor chambers and filled with the receptor phase. Phosphate buffer saline (PBS), pH 7.4 was used as the receptor medium. The small concentration of sodium azide (0.0025% w/v) was added to prevent any microbial growth [20]. The entire setup was placed over magnetic stirrer, and the temperature was maintained at 37 *± *0.5°C. The skin sections were initially left in the Franz cells for 2 hours in order to facilitate hydration of the skin samples. After this period, 5 mL of the appropriate formulation was applied onto the surface of the skin. 1 mL of medium was collected from the receptor compartment at predetermined intervals over study period and replaced with the same amount of fresh buffer. The amount of permeated drug was measured using a UV-Visible spectrophotometer (Thermo Spectronic UV-1, USA) by measuring absorbance at *λ*_*Max*⁡_ 248 nm.

### 2.6. Skin Permeation Data Analysis

#### 2.6.1. Permeation Flux

The amount of ondansetron HCl from various microemulsions was permeated while porcine skin was plotted against the function of time. The slope and intercept of the linear portion of plots were derived by regression. The permeation fluxes for each microemulsions were calculated as the slope divided by the skin surface area [15, 21].

*J*_*ss*_ = (*dQ*/*dt*)_*ss*_ · 1/*A*, where *J*_*ss*_  is the steady-state permeation flux (*μ*g/cm^2^/hour), *A *is the area of skin tissue (cm^2^) through which drug permeation takes place, and  (*dQ*/*dt*)_*ss*_  is the amount of drug passing through the skin per unit time at a steady state (*μ*g/hour).

#### 2.6.2. Kinetics

The data of *in vitro* ondansetron HCl permeation from various ondansetron HCl microemulsions through porcine skins were evaluated kinetically using various mathematical models like zero order, first order, Higuchi, and Korsmeyer-Peppas model equations [15].

Zero order kinetics: *F* = *K*_*o*_ *t*, where *F* represents the fraction of drug released in time *t* and *K*_*o*_ is the zero order release constant.

First order kinetics: ln (1 − *F*) = − *K*_1_ *t*, where *F* represents the fraction of drug released in time *t* and *K*_1_ is the first-order release constant.

Higuchi model: *F* = *K*_*H*_ *t* ^1/2^, where *F* represents the fraction of drug released in time *t* and *K*_*H*_  is the Higuchi dissolution constant. 

Korsmeyer-Peppas model: *F* = *K*_*p*_ *t* ^*n*^, where *F* represents the fraction of drug released in time *t* and *K*_*p*_ is the Korsmeyer-Peppas release rate constant, and *n* is the diffusion exponent.

### 2.7. Stability Studies 

#### 2.7.1. Centrifuge Stress Test

Various ondansetron HCl microemulsions were evaluated by centrifugation (Remi Motors, India) at 1250 rpm for a period of 5 hours and then, they were examined for any phase separation [22]. 

#### 2.7.2. Freeze-Thaw Cycles (FTC) Test

Various ondansetron HCl microemulsions were submitted to a total of 3 complete cycles; each cycle consisting of 24 hours at 25°C followed by 24 hours at −5°C [13].

### 2.8. Droplet Size, Polydispersity Index and Zeta Potential Determination. 

Droplet size, polydispersity index and zeta potential of the best formulation were determined using a laser scattering particle size analyzer (MALVERN ZETASIZER, MAL500999). 0.1 mL of the microemulsion was diluted to 10 mL of doubled distilled water to prepare samples for study.

## 3. Results and Discussion

### 3.1. Pseudoternary Phase Diagram. 

A phase behaviour investigation to develop microemulsion systems is the suitable approach of determining the water phase, oil phase, surfactant/cosurfactant concentrations. Therefore, pseudoternary phase diagrams of investigated microemulsion systems were constructed to determine compositions of microemulsions. The microemulsion regions in pseudoternary phase diagrams to determine the composition of an oil phase containing oleic acid or isopropyl myristate, an aqueous phase, and a surfactant/cosurfactant (3 : 1) phase containing Tween 80 as surfactant and isopropyl alcohol as cosurfactant for the formulation of HCl microemulsions at room temperature, which were represented in Figures 1(a) and 1(b), as non-darkened area. From the microemulsion regions in the pseudoternary phase diagrams, eight formulas were selected for the development of ondansetron HCl microemulsions (Table 1). Using the composition of selected microemulsion formulas, ondansetron HCl microemulsions were formulated and investigated.

### 3.2. * In Vitro * Skin Permeation. 

**Figure 2:**
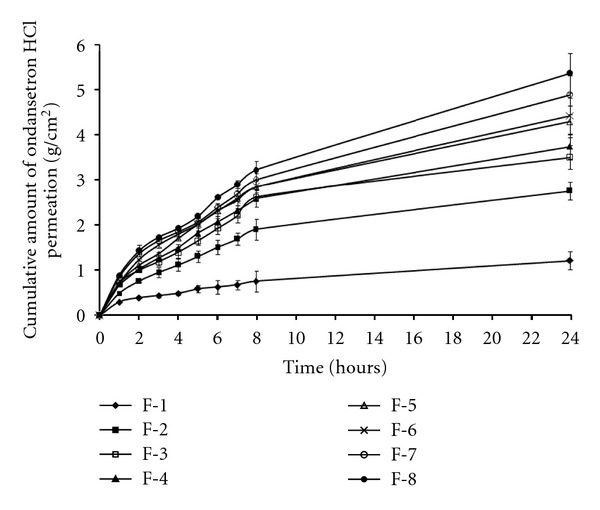
*In vitro *ondansetron HCl permeation profile through porcine skin per unit area from ondansetron HCl microemulsions (mean ± standard error, *n* = 3).

These ondansetron HCl microemulsions were investigated for *in vitro *skin permeation through excised porcine skin. The *in vitro *skin permeation from these formulated microemulsions was sustained over 24 hours (Figure 2). The amount of ondansetron HCl permeated over 24 hours period was plotted against the function of time. The permeation fluxes (*μ*g/cm^2^/hour) for all these microemulsions through the excised porcine skin were determined and presented in Table 1. This result showed higher permeation profile (with the highest permeation flux of 0.284 ± 0.003 *μ*g/cm^2^/hour) for microemulsion F-8, which contained 10% of isopropyl myristate as oil phase, 8% of aqueous phase, and 82% of surfactant phase containing Tween 80 and isopropyl alcohol, 3 : 1. The permeation flux of ondansetron HCl microemulsions containing oleic acid as oil phase was comparatively lower than that of microemulsions containing isopropyl myristate as oil phase, when all other excipients were the same. It was also apparent that the ondansetron HCl permeation was increased with the increase in the amount of surfactant phase and aqueous phase in their composition. This could be attributed to skin permeation enhancement capacity by the surfactants. Surfactants can loosen or fluidize the lipid matrix of the stratum corneum—the principal diffusional barrier of the skin—and act as skin permeation enhancer [23]. In addition, other components such as isopropyl myristate or oleic acid, which were used as oil phase in these formulated microemulsions, have the capacity as skin permeation enhancers and they could add the skin permeation enhancement of ondansetron HCl from formulated microemulsions.

In order to predict and correlate the *in vitro *ondansetron HCl permeation behavior from ondansetron HCl microemulsions through excised porcine skin, the *in vitro *permeation data were evaluated kinetically using various mathematical models like zero-order, first-order, Higuchi, and Korsmeyer-Peppas model. The results of the curve fitting into these above-mentioned models indicate the *in vitro *ondansetron HCl permeation behavior of ondansetron HCl microemulsions (F-1 to F-8) (Table 2). When respective correlation coefficients were compared, it was found that all these microemulsions followed the Korsmeyer-Peppas model (*R* ^2^ = 0.971 to 0.998), over a period of 24 hours as a best fit amongst all other models investigated. Again, the Korsmeyer-Peppas model was employed in the *in vitro *ondansetron HCl permeation behavior analysis of these formulations to find out permeation mechanisms: Fickian (nonsteady) diffusional release when *n* ≤ 0.5, case-II transport (zero-order) when *n* ≥ 1, and non-Fickian “anomalous” release when the value of n is between 0.5 and 1 [24]. The determined values of diffusion exponent (*n*) ranged between 0.529 and 0.602 (Table 2). These results indicated that the drug permeation from these ondansetron HCl microemulsions followed the non-Fickian, “anomalous” mechanism.

### 3.3. Stability

The stability of all these formulated ondansetron HCl microemulsions was studied by performing centrifuge stress test, and freeze-thaw cycles (FTC) test. After 5 hours of centrifugation at 1250 rpm and 3 complete freeze thaw cycles (each cycle consisting of 24 hours at 25°C followed by 24 hours at −5°C), all ondansetron HCl microemulsions were found stable as there were no sign of phase separation. 

### 3.4. Droplet Size, Polydispersity Index, and Zeta Potential Determination

**Figure 3:**
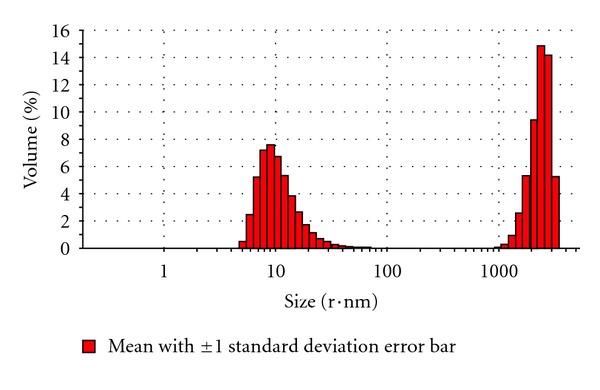
The droplet volume distribution curve of ondansetron HCl microemulsion formulation F-8 (10% of isopropyl myristate as oil phase, 8% of aqueous phase, and 82% of surfactant phase containing Tween 80 and isopropyl alcohol, 3 : 1).

**Figure 4:**
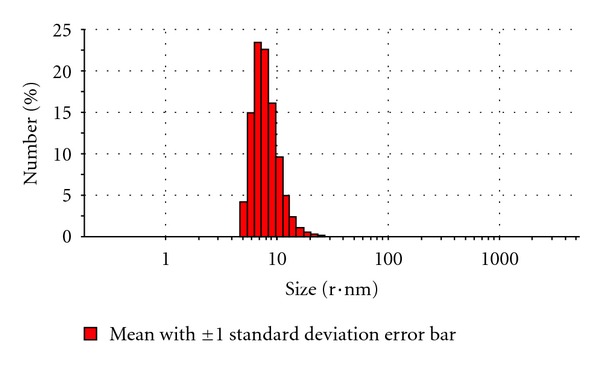
The droplet number distribution curve of ondansetron HCl microemulsion formulation F-8 (10% of isopropyl myristate as oil phase, 8% of aqueous phase, and 82% of surfactant phase containing Tween 80 and isopropyl alcohol, 3 : 1).

From the above results, ondansetron HCl microemulsion formulation F-8 (10% of isopropyl myristate as oil phase, 8% of aqueous phase, and 82% of surfactant phase containing Tween 80 and isopropyl alcohol, 3 : 1) was selected as best formulation based on its higher permeation flux through the excised porcine skin than other microemulsions formulated in this investigation. The ondansetron HCl microemulsion F-8 was further studied for determination of droplet size, polydispersity index, and zeta potential. The droplet size of the ondansetron HCl microemulsion F-8 was measured by a laser scattering particle analyzer Malvern Zetasizer (MAL 500999). The average droplet diameter (z-average diameter) of best microemulsion formulation was 427.53 nm. The droplet volume distribution curves and number distribution curves of ondansetron HCl microemulsion F-8 were presented in Figures 3 and 4, respectively. Due to its small droplet size, its surface areas were assumed high. Therefore, droplets of microemulsion settled down to close contact with the skin providing high concentration gradient and improved permeation of drug from microemulsions. The zeta potential of microemulsion F-8 was −11.80 mV. The skin has also slight negative charge. Therefore, the negative zeta potential of microemulsion F-8 might cause an influence in improved drug permeation through the skin due to electrostatic repulsion between the same charge of the skin surface and the microemulsion [15]. The polydispersity index has been found to be 0.35, which means that the globules are homogeneously distributed.

## 4. Conclusion

Ondansetron HCl microemulsions were developed using oleic acid or isopropyl myristate as the oil phase, Tween 80 as the surfactant, and isopropyl alcohol as the cosurfactant. All these microemulsions showed sustained drug permeations through the excised porcine skin and followed the Korsmeyer-Peppas model with non-Fickian, “anomalous” mechanism over a period of 24 hours. Among them, this highest permeation flux of 0.284 ± 0.003 *μ*g/cm^2^/hour was measured in case of ondansetron HCl microemulsion F-8 (contained 10% of isopropyl myristate as oil phase, 8% of aqueous phase, and 82% of surfactant phase containing Tween 80 and isopropyl alcohol, 3 : 1). The average droplet diameter, Zeta potential, and polydispersity index of the ondansetron HCl microemulsion F-8 were found as 427.53 nm, −11.80 mV, and 0.35, respectively. Overall, these results showed the promise of ondansetron HCl microemulsions for transdermal delivery in the treatment and/or prophylaxis of postoperative or chemotherapy- or radiotherapy-induced emesis with improved patient compliance.

## Figures and Tables

**Table 1:** Compositions of various ondansetron HCl microemulsions and skin permeation fluxes (*μ*g/cm^2^/hr) through excised porcine skin^a^.

Codes	Oil (%)	Surfactant and cosurfactant (3 : 1)	Water (%)	Permeation flux
		(Tween 80 and isopropyl alcohol, %)		(*μ*g/cm^2^/hour)^b^
	Oleic acid			
F-1	25	60	15	0.090 ± 0.001
F-2	20	65	15	0.146 ± 0.001
F-3	15	75	10	0.186 ± 0.002
F-4	10	82	8	0.199 ± 0.002
	Isopropyl myristate			
F-5	25	60	15	0.229 ± 0.002
F-6	20	65	15	0.233 ± 0.002
F-7	15	75	10	0.257 ± 0.003
F-8	10	82	8	0.284 ± 0.003

^a^All microemulsions contained 20 mg of ondansetron HCl.

^b^Mean ± S.D., *n* = 3.

**Table 2:** Results of curve fitting of the *in vitro *skin permeation data of various ondansetron HCl microemulsions.

Codes	Correlation coefficient (*R* ^2^)				Diffusion coefficient (*n*)
	Zero-order	First-order	Higuchi	Korsmeyer-Peppas
F-1	0.472	0.967	0.996	0.998	0.529
F-2	0.527	0.889	0.982	0.984	0.574
F-3	0.481	0.863	0.968	0.971	0.587
F-4	0.515	0.885	0.981	0.984	0.574
F-5	0.535	0.897	0.983	0.987	0.602
F-6	0.571	0.921	0.991	0.985	0.587
F-7	0.631	0.949	0.991	0.994	0.554
F-8	0.673	0.946	0.985	0.992	0.579
